# Supplementary material for: Circulatory Metabolite Ratios as Indicators of Lifestyle Risk Factors Based on a Greek NAFLD Case–Control Study
Source: Nutrients. 2024 Apr 21;16(8):1235. doi: 10.3390/nu16081235 (PMC11055137; doi:10.3390/nu16081235)
Supplement: Supplementary file 1 [file nutrients-16-01235-s001.zip › nutrients-2874904-supplementary.pdf]

Supplementary Figure S1 a. Permutation testing with 999 permutations. b. ROC curves

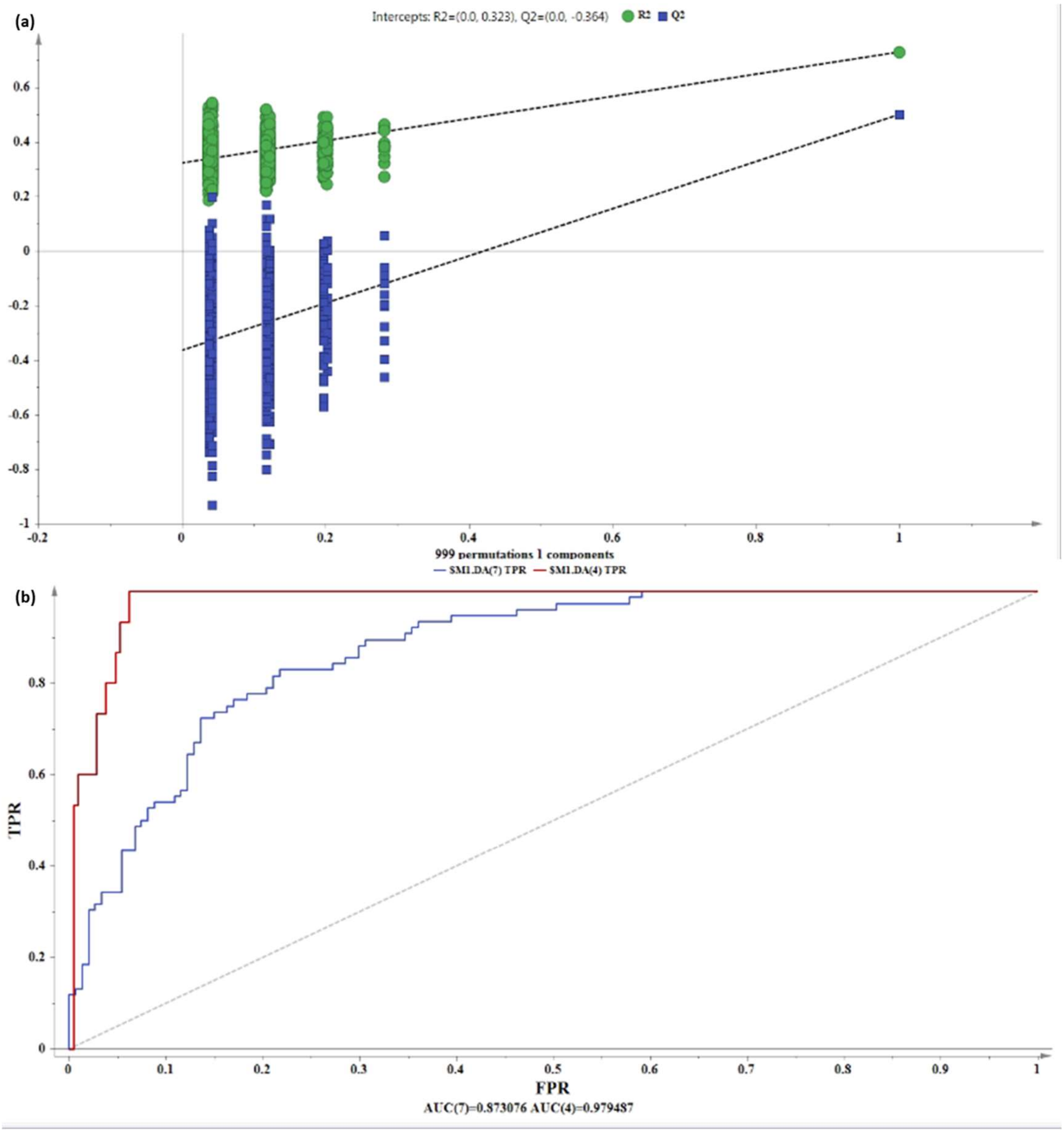

Supplementary Figure S2 *Box plots and ROC curves (*0.8> AUROC>0.7 *)for each metabolite differentially abundant between lifestyle 3-4 and 7-8*

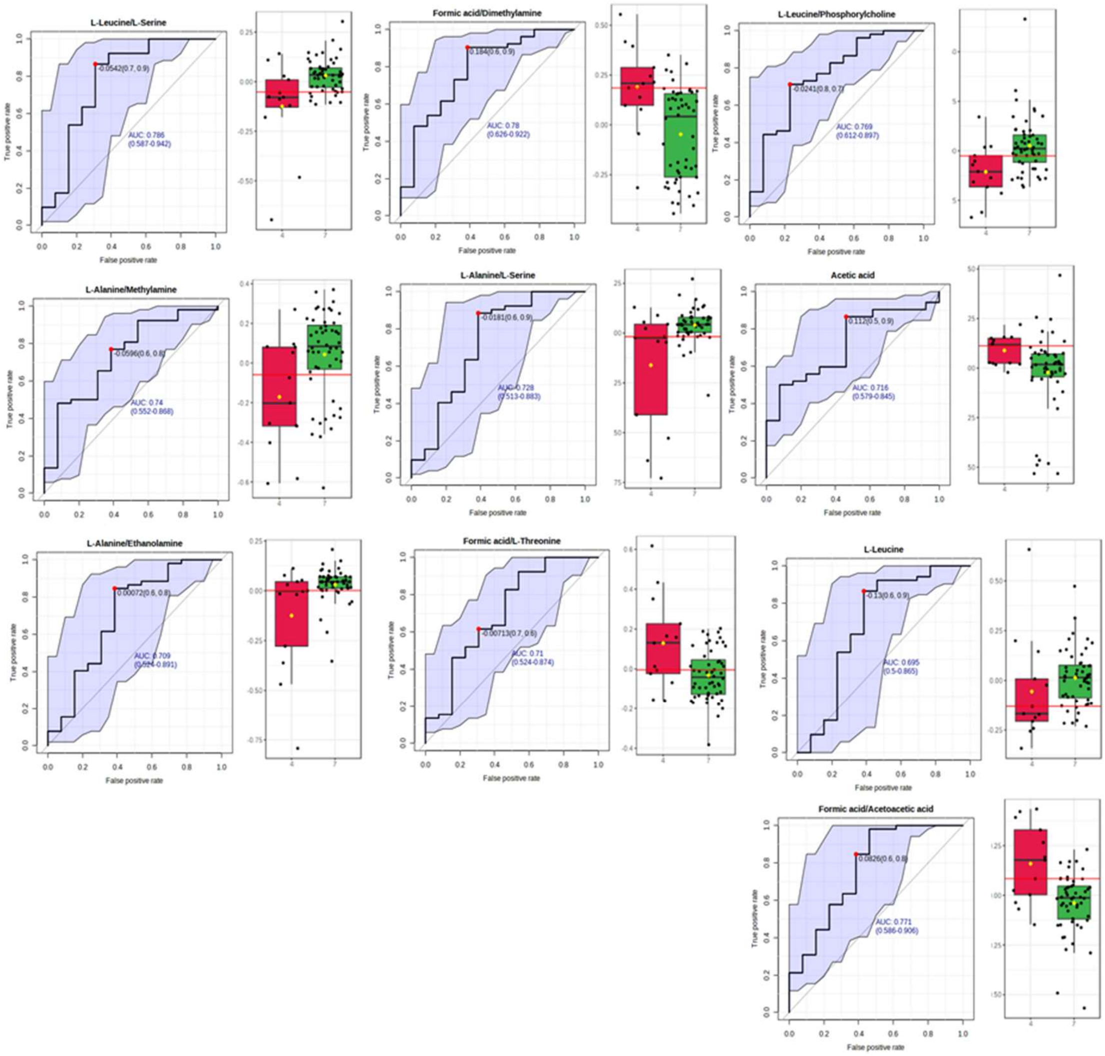

Supplementary Table S1 A summary of the primary disturbed statistically significant pathways (p<0.05),

Pathway Name	Match Status	p	-log(p)	Holm p	FDR	Impact
Aminoacyl-tRNA biosynthesis	3/48	1,09E+00	39.616	0.009177	0.009177	0.0
Phenylalanine, tyrosine and tryptophan biosynthesis	1/4	0.010293	19.875	0.85429	0.42978	0.5
Valine, leucine and isoleucine biosynthesis	1/8	0.020506	16.881	1.0	0.42978	0.0
Ubiquinone and other terpenoid-quinone biosynthesis	1/9	0.023046	16.374	1.0	0.42978	0.0
Phenylalanine metabolism	1/10	0.025582	15.921	1.0	0.42978	0.0

Supplementary Table S2 Metabolite enrichment analysis to uncover potential associations of lifestyle with other medical conditions

Metabolite Set	Total	Hits	Expect	P value	Holm P	FDR
DIFFERENT SEIZURE DISORDERS	24	3	0.2	4.3E-4	0.117	0.117
PHENYLKETONURIA	7	2	0.0585	0.00109	0.295	0.148
HEART FAILURE	10	2	0.0835	0.00231	0.625	0.157
REFRACTORY LOCALIZATION-RELATED EPILEPSY	10	2	0.0835	0.00231	0.625	0.157
ACUTE SEIZURES	14	2	0.117	0.00461	1.0	0.207
EARLY MARKERS OF MYOCARDIAL INJURY	14	2	0.117	0.00461	1.0	0.207
SCHIZOPHRENIA	26	2	0.217	0.0159	1.0	0.413
INFLAMMATORY DISEASES	2	1	0.0167	0.0166	1.0	0.413
LEIGH`S SYNDROME, SUBACUTE NECROTIZING ENCEPHALOPATHY, SNE	2	1	0.0167	0.0166	1.0	0.413
METHANOL POISONING	2	1	0.0167	0.0166	1.0	0.413
DENGUE FEVER	3	1	0.0251	0.0249	1.0	0.485
HAWKINSINURIA	3	1	0.0251	0.0249	1.0	0.485
PYRUVATE DEHYDROGENASE E3-BINDING PROTEIN DEFICIENCY	3	1	0.0251	0.0249	1.0	0.485
MYOCARDIAL INFARCTION	4	1	0.0334	0.0331	1.0	0.563
FRUCTOSE-1,6-DIPHOSPHATASE DEFICIENCY	5	1	0.0418	0.0412	1.0	0.563
N-ACETYLGLUTAMATE SYNTHETASE DEFICIENCY. NAGS DEFICIENCY	5	1	0.0418	0.0412	1.0	0.563
POST TRANSURETHRAL PROSTATIC RESECTION	5	1	0.0418	0.0412	1.0	0.563
TYROSINEMIA I	5	1	0.0418	0.0412	1.0	0.563
METABOLITES AFFECTED BY EXERCISE	5	1	0.0418	0.0412	1.0	0.563
